# Compressed models for co-reference resolution: enhancing efficiency with debiased word embeddings

**DOI:** 10.1038/s41598-023-45677-0

**Published:** 2023-10-28

**Authors:** Georgios Ioannides, Aishwarya Jadhav, Aditi Sharma, Samarth Navali, Alan W. Black

**Affiliations:** 1 Language Technologies Institute, Carnegie Mellon University, Pittsburgh, 15213 USA; 2 James Silberrad Brown Center for Artificial Intelligence, San Diego State University, San Diego, 92182 USA

**Keywords:** Engineering, Electrical and electronic engineering

## Abstract

This work presents a comprehensive approach to reduce bias in word embedding vectors and evaluate the impact on various Natural Language Processing (NLP) tasks. Two GloVe variations (840B and 50) are debiased by identifying the gender direction in the word embedding space and then removing or reducing the gender component from the embeddings of target words, while preserving useful semantic information. Their gender bias is assessed through the Word Embedding Association Test. The performance of co-reference resolution and text classification models trained on both original and debiased embeddings is evaluated in terms of accuracy. A compressed co-reference resolution model is examined to gauge the effectiveness of debiasing techniques on resource-efficient models. To the best of the authors’ knowledge, this is the first attempt to apply compression techniques to debiased models. By analyzing the context preservation of debiased embeddings using a Twitter misinformation dataset, this study contributes valuable insights into the practical implications of debiasing methods for real-world applications such as person profiling.

## Introduction

Co-reference resolution is a crucial task^1,2^ in the field of NLP, which involves identifying and grouping linguistic expressions that refer to the same real-world entity. This task plays a vital role in various downstream applications, such as question answering, text summarization, machine translation, and particularly in Information Extraction Systems, where co-reference resolution is a critical component. Co-reference relations establish an identity of reference between two textual elements, known as markables, and are essential for discourse analysis and language understanding in general. While the methods used in this study are applicable to any case of co-reference resolution, due to the nature of our data we specifically focus on gender co-reference resolution, which is of particular interest due to its potential implications in human profiling and the need to mitigate biases in language models. Human profiling refers to the process of categorizing people based on their characteristics, such as gender, race, or age. When NLP systems inadvertently incorporate and perpetuate biases present in the training data, they can contribute to human profiling by reinforcing stereotypes and personal identifying information. By addressing gender co-reference resolution, we aim to better understand these biases^3^. In recent years, deep learning models such as LSTMs and transformers^4^ have achieved significant advancements across various NLP tasks, including co-reference resolution. These models are trained on data annotated with noun phrases linked by co-reference relations, enabling them to learn these relationships and apply them to new documents.

However, these models are often large and resource-intensive, occupying considerable memory and requiring extensive computational power. For practical applications, smaller models with comparable accuracy would be advantageous, as they demand fewer computational resources and enable faster inference. The considerable resource requirements of large models not only increase training time, but also have a profound environmental impact due to the continuous operation of energy-intensive systems. In addition to being unfavorable in terms of latency and environmental impact, they might pose privacy issues when it comes to embedded systems (e.g., mobile phones), since data collected have to be processed centralized in case the size of such models exceeds the local memory constraints. Also, a good practise for achieving good generalization is to use the smallest dataset possible that fits the data. However, it is not always obvious what specific size is optimal.

Consequently, there is a growing need to develop lightweight models that efficiently disambiguate noun phrases during inference, minimizing both computational costs and environmental impact, while also ensuring privacy and good generalization. Developing such models will contribute to more sustainable and accessible NLP systems that can be employed across a broader range of applications and devices.

The stereotypes and biases present in NLP models can have real-world consequences in the context of privacy, human profiling, and security. These consequences may manifest in various forms, affecting people’s lives and exacerbating existing inequalities. For instance, in text classification, businesses and governments may use this analysis to gauge public opinion on specific topics or products. If the NLP models behind these tools are biased, they might misinterpret the context of specific demographic groups, leading to misguided decisions based on inaccurate or incomplete information. In surveillance and security, NLP models may be employed in surveillance systems to identify potential threats or criminal activities based on text analysis. Biases in these models could result in false positives or negatives, unfairly targeting or overlooking certain individuals or groups based on their demographics.

Mitigating biases in NLP models is indeed a challenging task, and identifying these biases is often even more difficult. These biases typically stem from the datasets used for training, reflecting the biases inherent in the human creators of the data. These biases can manifest in the results of downstream tasks and may also emerge when a specific gender is underrepresented in the datasets.

One approach to address this issue is to augment the dataset with an auxiliary dataset, as demonstrated in the work of Zhao et al.^5^. Training models on both the original and auxiliary datasets can effectively mitigate biases in the system. Therefore, it is crucial to investigate and address gender bias when employing models for co-reference resolution.

In NLP, the initial step (known the pre-processing stage) involves converting text into corresponding word embeddings (also called word representations). When biases are present in the dataset, they can become easily discernible in the word embedding vector space. These biases are often more prominently visible in downstream tasks (which is the step that follows the pre-processing stage), and become increasingly challenging to mitigate as they propagate further from the word embeddings.

Our work aims to address bias at the root level, specifically at the embedding level. We focus on the GloVe embeddings^6^ as an example, debiasing them and training models before and after debiasing, as well as before and after compression, using the debiased embeddings. We then examine the discrepancies in accuracy rate performance. Lastly, to assess the contextual preservation of the word embeddings post-debiasing, we evaluate the original and debiased word embeddings on a text classification task and report discrepancies in accuracy rates in order to examine any potential performance degradation.

In this study, we concentrate on gender bias and its impact on co-reference resolution systems^5,7^. Datasets used for this task employ templates to generate new samples by creating gender anti-stereotypical examples and subsequently train models on these datasets. They also compile a list of gender stereotypes and identify them within the datasets. Our *main contributions* are as follows:

- Debiasing of GloVe word embedding vectors (840B and 50) for the first time using HardWEAT and SoftWEAT methods (in the context of co-reference resolution) using the Word Embedding Association Test (WEAT) as the evaluation metric.
- Compression of co-reference resolution models using both original and debiased word embedding vectors achieving competitive performance measured by the accuracy rate.
- Assessment of context preservation in the debiased vectors performing text classification on both original and debiased word embedding vectors, while using accuracy rate as the evaluation metric.

## Related work

### Co-reference resolution

Linguistics-based approaches for the related problem of pronoun resolution have been explored since the 1980s^8,9^. The machine learning community has investigated the problem of co-reference resolution since the 1990s, employing both model-based and corpus-based approaches. Initial methodologies^10^ redefined the task as a pairwise classification problem and proposed a decision-tree approach for co-reference resolution, which was subsequently modified and extended by others^11,12^. Clustering algorithms have also been utilized for co-reference resolution by representing noun phrases as feature vectors that can be grouped together^13^. In contrast to pairwise classification, supervised clustering learns a similarity measure to generate clusters^14^. Other methods applied to this task include conditional random fields^15^ and co-training^16^. Other machine learning approaches were examined by^17^, which used annotated co-reference chains to generate additional co-reference data.

The task of co-reference resolution inherently involves identifying the referents of pronouns and noun phrases, which often carry gender information. The biases present in word embeddings can adversely affect the performance of co-reference resolution systems, leading to incorrect or biased resolutions. For instance, a biased system might incorrectly resolve the pronoun “she” to a stereotypically female profession rather than the correct referent in a given text. Therefore, debiasing word embeddings is crucial to enhance the fairness and accuracy of co-reference resolution systems. Our work aims to address this issue by employing debiasing techniques on word embeddings, which are then utilized in co-reference resolution tasks. By reducing gender bias in word embeddings, we strive to mitigate the propagation of such biases in co-reference resolution outcomes as well usage of these debiased word embeddings in other downstream NLP tasks (e.g. sentiment analysis, text classification etc.) while maintaining contextual meaning.

### Model compression

Recent advances in NLP have emphasized model compression, which aims to reduce the size and computational cost of an overparametrized model while retaining as much performance as possible. Scalar Quantization (or Precision Quantization)^18^ reduces the number of bits used to represent a weight value in a model. For simplicity reasons, Scalar Optimization is outside the scope of this work since it is hardware-specific (e.g. using different primitives for x86 and ARM architectures). Vector Quantization (or Product Quantization) reduces drastically the set of accessible weights by representing each group of weights by its centroid point, as in k-means and some other clustering algorithms. In general, model compression employs techniques such as Pruning, Quantization, Knowledge Distillation, Parameter Sharing, Tensor Decomposition, and Sub-quadratic Transformer-based methods^19^.

Model compression can be particularly relevant in the context of debiasing, as it may inadvertently affect the biases present in a model. For instance, pruning insignificant weights or quantizing the weights might alter the representation of gendered terms or biased associations within the model, thereby affecting the biases inherent in the model. Moreover, the compression of models could potentially lead to the retention or even amplification of biases if not handled carefully. Our work explores the intersection of model compression and debiasing, investigating how debiased word embeddings can be incorporated into compressed models to mitigate biases, particularly in co-reference resolution tasks. This exploration is crucial as not only does it address the computational efficiency of NLP models at the inference stage, but it also addresses their fairness and accuracy in tasks sensitive to biases.

More specifically, Pruning^3^ involves setting insignificant weights in the model to zero, as these weights often do not contribute meaningfully to the final task^20^, which might potentially lead to degradation of generalization. For transformer models, irrelevant attention heads are pruned^21^. Pruning is generally considered to be more important than quantization because it can result in much higher compression rates without significant loss in accuracy. Also, sparsity can also accelerate inference time by reducing the number of multiplications required.

In contrast, Quantization^22^ may result in a loss of accuracy, especially for complex networks, and it may require additional calibration steps to optimize the quantization parameters. This is because Vector Quantization forces the weights and activations to fit into a fixed number of discrete values, which may not be optimal for representing the underlying data distribution. Moreover, Vector Quantization requires a careful selection of the number of discrete values or centroids to use for quantization. Selecting too few centroids can result in a large quantization error, while selecting too many centroids can increase the memory requirement and reduce the compression rate. The selection of centroids can also be computationally expensive, especially for large neural networks. However, Pruning does not require a fixed number of centroids, and despite its simplicity, it can preserve the important information in the model more than Quantization does, while also removing the redundant or unimportant network connections. Also, the simplicity of Pruning makes it easier to implement and automate.

Knowledge Distillation is a widely-used approach for obtaining smaller models in NLP, particularly for transformer models. The primary concept involves training a large model initially, followed by training a smaller model that mimics the larger model’s output^23,24^. Techniques like the one by Tang et al.^25^ distill knowledge from BERT into simpler models such as LSTM. Methods like the ones by Lan et al.^26^ reduce the size of transformer models by sharing parameters, ensuring they maintain the same value. Beltagy et al.^27^ proposed making the full self-attention matrix in transformers sparse, outperforming RoBERTa^28^ on various long document downstream tasks, including co-reference resolution.

### Bias analysis and debiasing

Bias in NLP is a research area that has been widely explored for tasks like language modeling^29^, co-reference resolution^30^, machine translation^31^ and sentiment analysis^32^. Various bias mitigation or debiasing techniques for co-reference resolution have evolved over the years, with many of them focusing on gender bias. Bolukbasi et al.^33^ explored word embedding debiasing in which vector representations of the gender are identified and compensated for deviation. Zhao et al.^5^ used vector space manipulation along with data augmentation by gender swapping and introduced a new benchmark (WinoBias) for gender bias focused co-reference resolution. Zhao et al.^34^ used data augmentation and embedding neutralization methods to mitigate gender bias in word embeddings. Zhao et al.^30^ proposed a novel training approach for learning gender-neutral word embeddings by attribute protection. Based on this approach, they generate a gender-neutral version of GloVE (GN-GloVe) and empirically show that GN-GloVe successfully separates gender information without compromising on embedding model functionality.

### Bias in compressed models

Xu and Hu^35^ showed that there is reduction of toxicity and bias in compressed GPT2 model compressed via Knowledge Distillation. In computer vision models, Quantization and Pruning methods have compromised fairness because performance of samples with under-represented features is sacrificed after compression^36,37^. However, to the best of the authors’ knowledge, there has been no study which operates on the domain of enhancing performance with debiased word embeddings in compressed models for co-reference resolution.

## Methods

**Figure 1:**
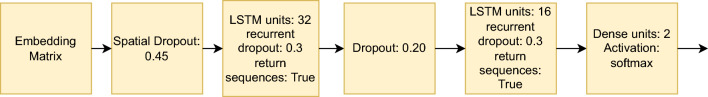
Proposed text classification system architecture.

These word embeddings (i.e. the GloVe embeddings) can be represented mathematically as $X \in {\textbf {R}}^{N \times d}$ where each $x_i$ is a *d*-dimensional vector. The architecture of the Text Classification system can be found in Fig. 1. This model architecture is the derivation of hyper-parameter tuning—varying the number of LSTM layers.

We look at two variants of debiasing, and evaluate the word embeddings based on that, namely *HardWEAT* and *SoftWEAT*. The methodologies are comprehensively delineated by Popovic et al.^38^. Both of these embeddings are post-processing steps. Both reply on identifying the gender bias subspace B via Principal Component Analysis computed on gender word pair differences.

In particular, HardWEAT debiasing employs a neutralizing technique that removes all non-gender words N from a gender subspace by deducting from vectors their bias subspace projection. Subsequently, an equalize operation positions opposite gender pair words to share the same angle with neutral words. In contrast, SoftWEAT. Debiasing enables more gradual bias removal by utilizing a tuning parameter $\lambda$. An embedding is being transformed by optimizing a transformation matrix $T \in \textbf{R}^{d \times d}$ in Eq. (1):

1 $$\begin{aligned} \min _T {||{(TW)}^T (TW)-W^T W||} ^{2} _F + \lambda {||{(TN)}^T (TB) ||} ^ {2} _F \end{aligned}$$

### Proposed approaches

The proposed approach consists of the following steps:

1. Collection and bebiasing of GloVe word embedding vectors: Two variations of GloVe word embedding vectors, i.e., 840B and 50, are collected. These vectors are debiased for gender using the HardWEAT and SoftWEAT ($\lambda =0.5$) methods^38^. A list of male and female words is used to perform debiasing. The debiased vectors are evaluated for gender bias using the Word Embedding Association Test.
2. Training and evaluation of co-reference resolution model: A co-reference resolution model is trained using both the original and debiased word embedding vectors. The debiased vectors are obtained using SoftWEAT and HardWEAT methods. The accuracy rate is used to evaluate the performance of the model.
3. Training and evaluation of compressed co-reference resolution model: A co-reference resolution model is trained using both the original and debiased word embedding vectors (using SoftWEAT and HardWEAT methods), and then compressed using the methods outlined in later sections of the report. The accuracy rate is used to evaluate the performance of the compressed model.
4. Training and evaluation of text classification model: A text classification model is trained using both the original and debiased word embedding vectors (using SoftWEAT and HardWEAT methods). The Twitter misinformation dataset is used to evaluate the degree to which the context of the debiased word embedding vectors is preserved. The dataset’s categories have been consolidated into two primary groups: *true information* and *false information*. The “true information” category encompasses the original categories of *True Treatment*, *True Fact*, *Correction/Calling Out*, *Sarcasm/Satire*, and *True Public Health Response*. The “false information” category includes the original categories of *Conspiracy*, *Fake Cure*, *Fake Treatment*, *False Fact or Prevention*, and *False Public Health Response*. The accuracy rate is used as the evaluation metric.

**Figure 2:**
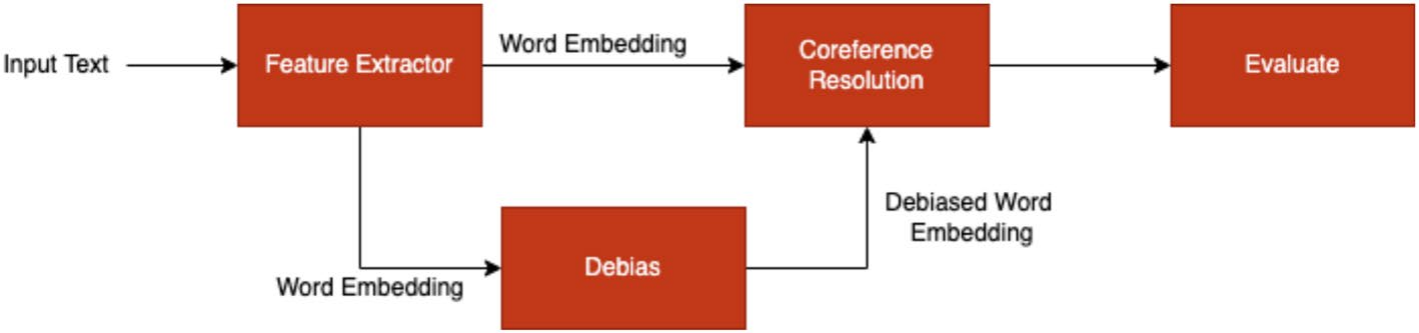
Proposed co-reference resolution evaluation pipeline.

The pipeline followed for steps 1–3 is illustrated in Fig. 2. We alter the Embedding Distribution to mitigate bias and reduce bias right at the beginning, that is at the embedding level itself. This is achieved by altering the spatial distribution of neighboring vectors which helps in achieving a bias-free setting while minimizing semantic offset

## Dataset

In this work the following datasets are used: (i) WinoBias^5^ and (ii) CMU-MisCOV19 dataset^39^. The WinoBias dataset evaluates gender bias in coreference resolution systems. It is structured around people entities referred by their occupations from a vocabulary of 40 occupations gathered from the US Department of Labor. It uses associated occupation statistics to determine what constitutes gender stereotypical roles It contains 3160 sentences, testing if systems link gendered pronouns to stereotypical occupations accurately. The dataset aims to highlight and address potential gender bias in co-reference resolution, providing a benchmark for system evaluation. Pronouns of occupations dominated by the gender of the pronoun are called pro-stereotyped and occupations not dominated by the gender of the pronoun are called anti-stereotyped. The CMU-MisCOV19 dataset, described in the provided paper, comprises 4573 annotated tweets to characterize COVID-19 misinformation on Twitter. It categorizes users as misinformed or informed and tweets into 17 categories like “True Treatment,” “Fake Cure,” etc., aiming to analyze the spread and correction of misinformation.

## Results and discussion

### Evaluation setup

We use a couple of metrics which help in identifying gender bias in our co-reference systems.

#### Word embedding association test

In this work we measure the bias of the system at the embedding level, and use these embeddings to perform co-reference resolution. To help in evaluating these scores we employ the WEAT. We use the WEAT to obtain a measure of the bias present in the embeddings. The WEAT takes into account two sets of targeted words(gendered names or nouns), $T_1$ and $T_2$ and two sets of attribute words $A_1$ and $A_2$ (words related to occupation), this test provides an objective score based on a permutation. The null hypothesis of this test would be that the relative association of target sets’ words to the attribute sets’ words are equally strong. The score depends on the association of the target words with the set of attribute words. The higher the score the more is the association of $T_1$ with $A_1$ and $T_2$ with $A_2$. On the other hand the more negative the value, the more is the association of $T_1$ with $A_2$ and of $T_2$ with $A_1$. The ideal score is 0 which shows that neither $T_1$ nor $T_2$ is associated with either $A_1$ or $A_2$ and that indicates the absence of bias in the word embeddings. The WEAT expects a query of the kind ($T_1$, $T_2$, $A_1$, $A_2$), the first defintion done by WEAT is as defined below in Eq. (2),

2 $$\begin{aligned} d(w, A_1, A_2) = (mean_{w \epsilon {A_1}} cos(w,x)) - (mean_{w \epsilon {A_2}} cos(w,x)) \end{aligned}$$

where cos(w, x) is the cosine similarity of the word embedding vectors. The WEAT metric for a query of the form ($T_1$, $T_2$, $A_1$, $A_2$) is defined as in Eq. (3):

3 $$\begin{aligned} F_{WEAT} (M, Q)&= \sum _{w \epsilon {T_1}} d(w, {A_1}, {A_2}) - \sum _{w \epsilon {T_2}} d(w, {A_1}, {A_2}) \end{aligned}$$

#### Accuracy

We also use accuracy as a metric to evaluate the performance of our models. For every sentence, we retrieve the ground truth and the prediction. These are represented as lists containing an occupation (for example, ‘the manager’, ‘the teacher’, ‘the farmer’) and a corresponding gendered word (for example, ‘he’, ‘she’, ‘him’, ‘her’). Accuracy is simply defined as the ratio of correct predictions (where the prediction matches the ground truth) to total number of sentences.

### Establishing baselines

In this study, we examine the impact of compression and debiasing techniques on word embeddings in relation to the bias present within co-reference resolution models. Consequently, our baseline consists of a co-reference resolution model that does not employ debiased word embeddings or compression methods.

The baseline co-reference resolution model is based on the work by Lee et al.^40^, which introduces a higher-order co-reference resolution approach utilizing coarse-to-fine inference. This model leverages an antecedent distribution derived from a span-ranking architecture as an attention mechanism, allowing for continuous refinement of span representations. As a result, the model can softly consider multiple hops in the predicted clusters. While this process can be computationally intensive, the coarse-to-fine approach incorporates a less accurate yet more efficient bi-linear factor. This enables effective pruning without compromising performance. The baseline scores can be found in the following subsections.

### Debiased word embeddings

The word embeddings are debiased and evaluated according to gender following the approaches used by Popović et al.^38^. In the seminal work by Popović et al.^38^, the parameter $\lambda$ is delineated, possessing a range between 0 and 1. A $\lambda$ value of 0 signifies the utilization of HardWEAT, while a value greater than 0 indicates the application of SoftWEAT. Upon reviewing Table 2 in the referenced paper^38^, it is discernible that the optimal performance on GloVe embeddings is achieved with a $\lambda$ value consistently below 0.6, which informed our choice of 0.5 for the $\lambda$ parameter in our methodology. A set of male and females words have been selected. These words are then selected from the word embeddings and are then debiased using SoftWEAT and HardWEAT. More specifically, the word embeddings used are *GloVe 840B* which contain a total of 2,195,487 gender-neutral words out of the total 2,195,988 words, and *GloVe 50* which contain a total of 454,643 gender-neutral words out of the total 455,002 words in the vocabulary. Nonetheless, even a relatively small sample of non-gender-neutral words can have a significant influence on downstream tasks as it can be observed by the results in Tables 1 and 2. This highlights the importance of our work for real-world applications that commonly use such readily available word embeddings.

**Table 1:** Winobias co-reference resolution accuracy rate on original and debiased GloVe word embeddings.

Debiased GloVe embeddings	Anti-stereotype$\uparrow$		Pro-stereotype$\uparrow$	
	Type 1	Type 2	Type 1	Type 2
Original	0.346	0.73	**0.657**	0.848
SoftWEAT ($\lambda =0.5$)	0.359	0.677	0.492	0.684
HardWEAT	**0.462**	**0.753**	0.641	**0.866**

Significant values are in bold (best perfomance).

**Table 2:** Winobias test set performance on debiased GloVe word embedding vectors using compressed models.

Debiased GloVe word embedding vectors						
	Anti-stereotype			Pro-stereotype		
	THS	Accuracy rate $\uparrow$		THS	Accuracy rate$\uparrow$	
		Orig.	HardWEAT/SoftWEAT		Orig.	HardWEAT/SoftWEAT
Type 1	0.1	0.290	0.422/0.306	0.1	0.636	0.422/0.616
	0.02	0.348	**0.449**/0.359	0.02	**0.649**	0.449/0.639
Type 2	0.1	0.740	0.697/**0.758**	0.1	0.838	0.697/0.848
	0.02	0.732	0.682/**0.758**	0.02	0.848	0.682/**0.864**

Significant values are in bold (best perfomance).

The original GloVe word embedding vectors have a WEAT score (i.e. between the selected male and female words) of 0.40 in the case of the *840B* version of the word embedding vectors and 0.62 in the case of the *50* version of the word embedding vectors. Using the *SoftWEAT* method, this bias can be reduced to approximately half of the original WEAT bias and using *HardWEAT* completely eliminates any existing bias as estimated by the WEAT score. These results can be found in Tables 3 and 4 respectively.

**Table 3:** GloVe-300D word embedding vectors.

Debias method	WEAT score$\downarrow$
None	0.40
SoftWEAT ($\lambda =0.5$)	0.21
HardWEAT	**0.00**

Significant values are in bold (best perfomance).

**Table 4:** GloVe-50D word embedding vectors.

Debias method	WEAT score$\downarrow$
None	0.62
SoftWEAT ($\lambda =0.5$)	0.36
HardWEAT	**0.00**

Significant values are in bold (best perfomance).

### Co-reference resolution

For co-reference resolution, we employ the end-to-end co-reference resolution model with Coarse-to-fine inference^41^, trained on English OntoNotes 5.0. To assess the gender bias present in this model, we evaluate its performance on the WinoBIAS dataset (https://github.com/uclanlp/corefBias/tree/master/WinoBias/wino/data). The original co-reference resolution model’s performance is presented in Table 1, which considers only 50 antecedents per span.

The model employs 3 highway LSTMs and 2 types of GloVe embeddings:

GloVe-300D (http://downloads.cs.stanford.edu/nlp/data/glove.840B.300d.zip) for context and GloVe-50D for head embeddings. To mitigate the gender bias in these embeddings, we employ the SoftWEAT and HardWEAT debiasing methods as discussed in the previous section. The results obtained after debiasing both these embeddings are presented in Table 1. We observe that the HardWEAT method effectively reduces bias in all types of anti-stereotyped data and type-2 pro-stereotyped data.

### Model compression

To reduce the computational complexity of the co-reference resolution model described in the previous section, we employed a Pruning method. This technique does not require additional training, unlike the Knowledge Distillation method, and is therefore computationally efficient.

The Pruning process involves setting the parameters of a trained model to zero if their values fall below a certain threshold (THS). In our work, we set this threshold to 0.1 and 0.02, resulting in compression rates of 33.25% and 6.66% of the total weights, respectively. It should be noted that this compression technique does not include the compression of the GloVe embeddings, which we also utilized to reduce the model’s bias.

The compressed model can demonstrate improved speed in inference if the sparse tensor algorithm is employed, which we leave as a future direction of research. Table 2 illustrates the results of compressing the original end-to-end co-reference model at the two threshold values. We observed that the model with 33.25% compression (with 0.1 threshold) can actually reduce the bias in anti-stereotypical data.

We further evaluated the performance of the compressed co-reference resolution model using the HardWEAT and SoftWEAT debiased word embeddings. The results are presented in Table 2. Notably, the use of debiased word embeddings further reduces bias in the compressed co-reference resolution model.

To perform model compression, two settings were implemented and the results of each were recorded:

1. Setting ${\textbf {w}}=0$ when $\mid w \mid < 0.1$ resulting in 33.25% of the weights ${\textbf {w}}$ to be eliminated.
2. Setting ${\textbf {w}}=0$ when $\mid w \mid < 0.02$ resulting in 6.88% of the weights ${\textbf {w}}$ to be eliminated.

Referring to Table 2, there was in general a considerable accuracy deterioration when *HardWEAT* debiased word embedding vectors were used in model compression. However, using *SoftWEAT* debiased word embedding vectors, there was in general an accuracy rate increase which was even better in most cases than using the Original (Orig.) biased word embedding vectors. This implies, that with proper hyper-parameter tuning of the regularization parameter, $\lambda$, in *SoftWEAT*, it is possible to even improve model performance on the original word embedding vectors. This is possibly because the bias in the word vectors is partially removed, and therefore, it no longer affects the model accuracy as much, even though the dataset the model was trained on has been made intentionally gender biased.

### Text classification

Text classification focuses on the *true information* and *misinformation* categories. Each word in the GloVe word embedding vectors is modelled as $300 \times 1$ vector. Initially, the dataset vocabulary is collected and only the *top 5000* most frequent words are kept. This is not including stopwords, emojis and punctuation marks. The algorithm for constructing the Embedding Matrix can be found in Algorithm listing in the Supplementary Material.

Furthermore, an embedding matrix is constructed with dimensions of $5000 \times 300$, where the rows signify the number of distinct words in the vocabulary and the columns signify the dimensionality of each word vector. If a word in the top 5000 most frequent ones is not part of the GloVe word embedding vectors, then its word vector is initialized using the Xavier initialization method. The algorithm of this procedure can be found in listing.

Additionally, an initial learning rate of 0.1, an exponential decay learning rate schedule with a decay rate of 0.96 and total of 10,000 decay steps is utilized. The model was trained for a total of 15 epochs. The loss function used is Categorical Cross Entropy with the Adam optimizer. Using the original word embeddings gives the highest accuracy rate across both GloVe word embedding vector versions as it can be observed in Tables 5 and 6. Then, in seconds place comes *SoftWEAT* and finally *HardWEAT*.

**Table 5:** GloVe 840 word embedding vectors.

Debiased method	Accuracy rate$\uparrow$
None	**0.768**
SoftWEAT ($\lambda =0.5$)	0.745
HardWEAT	0.742

Significant values are in bold (best perfomance).

**Table 6:** GloVe 50 word embedding vectors.

Debiased method	Accuracy rate$\uparrow$
None	**0.768**
SoftWEAT ($\lambda =0.5$)	0.748
HardWEAT	0.748

Significant values are in bold (best perfomance).

Nonetheless, the debiased word embeddings do not exhibit a significant accuracy rate deterioration (worst case is − 2.6%) which comes at the benefit of *completely or partially* having gender debiased word embedding vectors. Therefore, the context of the original word embedding vectors is reserved in a very satisfactory level. However, it is desired to extend and evaluate the performance of the debiased word embeddings in text classification tasks to other larger and more corpora rich datasets.

In the text classification experiment, we aim to further assess the impact of debiased word embeddings on contextual meaning and their utility in broader language understanding tasks and domains. This additional experiment is included to examine the adaptability of the debiased embeddings at a paragraph level, which consists of one or more sentences. As articulated in the literature by Popovic et al.^5^, the text classification task serves as a valuable benchmark, testing whether the altered word embeddings still retain their efficacy for tasks beyond co-reference resolution. Our work, therefore, utilizes text classification as a “sanity check” to verify the versatility of the debiased word embeddings. The results obtained from this experiment contribute to a comprehensive understanding of the implications of debiasing word embeddings. While our primary focus is on the performance of co-reference resolution, it is noteworthy that, in the majority of cases, the post-debiasing word embeddings demonstrates an improvement in the co-reference resolution task while maintaining competitive performance in the text classification task. This dual evaluation provides evidence that debiased word embeddings can enhance performance in specific language understanding tasks without sacrificing their overall utility, reinforcing the practicality of the approach.

## Conclusion

A framework for word embedding vector debiasing is put into place and is used to debias GloVe word embedding vectors^6^ and is evaluated through the metric of Word Embedding Association Test. A co-reference resolution model is trained on the original (non-debiased) and debiased word embedding vectors and evaluated using the accuracy rate metric. Furthermore, this is repeated for a co-reference resolution model which was compressed and trained on the original and debiased word embedding vectors. Finally, a text classification model is trained to evaluate the contextual reservation of the original word embedding vectors after being debiased. Results suggest that with proper hyper-parameter tuning, a compressed co-reference model trained on debiased word embedding vectors can outperform uncompressed models trained on original word embedding vectors. This work constitutes as the first ever attempt that has been carried out in measuring the impact of compression in co-reference resolution in the context of estimating gender bias in NLP tasks.

### Supplementary Information

Supplementary Information.

## Data Availability

This work uses the following datasets: (i) WinoBias^5^ and (ii) COVID-19 Twitter Misinformation dataset^39^. The datasets generated and/or analysed during the current study are available in the *uclanlp/corefBias* repository, https://github.com/uclanlp/corefBias/tree/master/WinoBias/wino and *zenodo* repository, https://zenodo.org/record/4024154 respectively.
