# Supplementary material for: Compressed models for co-reference resolution: enhancing efficiency with debiased word embeddings
Source: Sci Rep. 2023 Oct 28;13:18510. doi: 10.1038/s41598-023-45677-0 (PMC10613201; doi:10.1038/s41598-023-45677-0)
Supplement: Supplementary file 1 — Supplementary Information. [file 41598_2023_45677_MOESM1_ESM.pdf]

Supplementary Material

Algorithm 1 Text Classification: Constructing Embedding Matrix

Input: GloVe word embeddings (Original or Debiased), Twitter dataset $\mathscr{D}$
Parameters: $V = 5000$, $D = 300$
Output: E
Initialize vocabulary $\mathscr{V}$ from Twitter dataset $\mathscr{D}$
Remove stopwords, emojis, and punctuation marks from $\mathscr{V}$
Select **top** V most frequent words from $\mathscr{V}$ as $\mathscr{V}'$
Initialize embedding matrix $E = \{E_w\}_{w=1}^{V} \in \mathscr{R}^{V \times D}$ with zeros
for each word $w \in \mathscr{V}'$ **do**
    if w in GloVe word embeddings **then**
        $E_w \leftarrow$ word embedding for w
    else
        $E_w \leftarrow$ Xavier initialization of vector with length D
    end if
end for
